# Integrated single-cell RNA sequencing and Bulk-RNA technologies reveal the immunological characteristics of lactylation related-genes in glioblastoma

**DOI:** 10.1371/journal.pone.0351849

**Published:** 2026-06-26

**Authors:** Biao Wang, Yangfang An, Yuansen Shu, Xiaoping Cheng, XuXiang Chen, Xiezhuo Zhang, Zhaorui Cheng

**Affiliations:** 1 Department of Neurology, Yiyang Central Hospital, Yiyang, Hunan, China; 2 Department of Emergency, The Eighth Affiliated Hospital of Sun Yat-sen University, Shenzhen, Guangdong, China; 3 The First Affiliated Hospital, Jiangxi Medical College, Nanchang University, Nanchang, Jiangxi, China; 4 Department of Neurosurgery, Yiyang Central Hospital, Yiyang, Hunan, China; Qatar Biomedical Research Institute, QATAR

## Abstract

**Objective:**

Glioblastoma (GBM) is the most aggressive type of intracranial malignant tumor, known for its extremely poor prognosis. Lactylation, a newly identified post-translational modification, has been linked to tumorigenesis, though its specific role in GBM remains unclear. This study aims to integrate single-cell RNA sequencing (scRNA-seq) and bulk RNA sequencing (RNA-seq) data to create a novel prognostic model for GBM, focusing on lactylation-related factors.

**Methods:**

We studied lactate metabolism genes as markers in GBM. We obtained bulk transcriptomic data from TCGA and the GSE141383 and GSE162631 cohorts in the GEO databases. We used the R package Seurat to analyze scRNA-seq data, CellChat for cell communication analysis, and AUCell to assess lactate metabolism gene set scores across cell types. We developed a prognostic model using machine learning algorithms and tested its efficacy across multiple cohorts. Additionally, we investigated differences in immune infiltration, predicted sensitivity, and other factors between high and low-risk groups. We validated the function of the key gene CD93 at the cellular level.

**Results:**

The scRNA-seq data identified nine major cell types in GBM, with FCGBP+ macrophages showing the highest score in the lactate metabolism gene set. Authors designed a model informed by machine learning pinpointed three key genes（CD93, FCER1G, and GRB2）and developed a model with optimal prognostic value across cohorts.The high-risk group presented significantly poorer clinical outcomes. Immune-related bioinformatic analysis revealed significant differences in immune cell infiltration and checkpoint gene expression between risk groups. High-risk patients demonstrated lower immune infiltration and higher immunosuppression, rendering them less suitable for immunotherapy. Predictive algorithms indicated that axitinib and imatinib could be potential therapeutic drugs for these high-risk patients. In GBM tissue and cells, CD93 expression was significantly elevated, identifying it as a key risk gene in this model. Inhibition of CD93 expression via siRNA significantly reduced the proliferation, invasion, and migration of U87 and U251 cells.

**Conclusion:**

In summary, we developed a novel characterization of lactylation-related clusters using single-cell sequencing technology. This study provided insights into the prognostic significance of lactate metabolism-related genes in GBM.

## Introduction

GBM is one of the most common primary intracranial tumors [1–2]. It is the most aggressive tumor of brain neuroglia and is the most commonly encountered primary intracranial tumor [3]. It is rapidly growing and densely infiltrating the brain and hence the deadliest tumor of the central nervous system [4]. Initial symptoms include nonspecific cognitive deficits and seizures [5]. Surgery, radiotherapy, and chemotherapy drugs are standard treatments. The median survival rate for GBM patients is under one year [6]. Recent work has identified several biomarkers that provide valuable information about the molecular subtypes of GBM, their prognosis and treatment responses [7]. However, due to its rapid growth and heterogeneous nature, it still remains difficult to treat for oncology. Finding therapeutic targets and developing prognostic risk models are critical for optimal clinical management of glioblastoma.

Since Warburg effect was introduced in 20th century, research has shown lactic acid plays a key role in initiation and progression of malignant tumors [8].Tumor tissues grow rapidly and require substantial nutrients and oxygen to sustain their proliferation. [9]. According to Warburg effect, tumor energy metabolism depends more on anaerobic glycolysis than oxidative phosphorylation even in the presence of oxygen. Increased lactic acid levels in tumor microenvironment lead to hypoxic microenvironment [10]. High levels in tumor microenvironment correlate to tumor growth, metastasis, and tumor cell evasion [11]. Tumor cells export large amounts of lactic acid to adjacent immune and stromal cells. Thus, lactic acid acts as mediator between endogenous metabolic processes and immune suppression [12]. She X [13] and colleagues showed that SETDB1 methylation inhibits interaction between MCT1 and Tollip and induces M2-like polarization of tumor-associated macrophages. Su J [14] and colleagues found that absence of lactic acid/GPR81 inhibits expression of chemokine CX3CL1, decreases Treg infiltration into tumor microenvironment (TME) and inhibits gastric cancer progression by improving CD8 + T cells function. The mechanisms of lactic acid production pathways or its transport to lower lactic acid concentrations is not fully understood.

We used scRNA-seq to investigate the role of lactylation in GBM cell diversity. We developed and validated a novel model using signature genes from FCGBP+ macrophages, which showed highest lactylation scores. We also studied the relationship between risk model and immune-infiltrating cells to identify potential therapeutic targets. We identified CD93 as important gene in the model and studied its effects on GBM cell function at cell level.

## Methods

### Ethics approval

All raw transcriptome data used in this study were downloaded from publicly available GEO and TCGA databases. No human or animal specimens were collected in the present work; therefore, ethical approval and informed consent were not required.

### Data collection

The data in this article were sourced from publicly accessible databases, specifically the TCGA (https://portal.gdc.cancer.gov/) and GEO (https://www.ncbi.nlm.nih.gov/geo/). The single-cell datasets used in this study, GSE141383 and GSE162631, include 13 GBM tissue samples and 4 control tissue samples. The bulk datasets are divided into two parts: 1) The training dataset for the diagnostic model comes from the TCGA-GBM cohort, comprising 5 control tissue samples and 168 GBM tissue samples, with survival information for 80 GBM patients; 2) The validation dataset for the diagnostic model is obtained from the CGGA-GBM database, which includes 137 GBM patients with survival information, and the GEO database (GSE83300), which contains 50 GBM patients with matched survival data.

### Data processing

For single-cell data analysis, Seurat version 4.2.2 was employed. Initially, quality control excluded cells with fewer than 200 or more than 5000 nFeature_RNA and those with a mitochondrial proportion over 10%. Data normalization and standardization were conducted using the SCTransform function. Dimensionality reduction and clustering were performed by selecting the top 15 principal components, and the RunUMAP function was used to generate results. The DoubletFinder tool removed doublets from the dataset. To address batch effects across datasets and samples, the Harmony algorithm was applied. Cell annotation was conducted using the SingleR method alongside existing literature. The FindAllMarkers function identified marker genes for each cell type. UMAP results were visualized using the ggplot2 package, and cell proportions were represented with the ggalluvial package. For bulk RNA data, normalization and standardization were performed using the limma package. Additionally, survival analysis and curve plotting were executed with the Survival and survminer packages.

### Ro/e analysis

The Ro/e ratio [15] illustrates the relationship between observed and expected cell counts for each cell cluster across different tissue types. Expected cell counts for each cell cluster and tissue type combination are derived from chi-square tests. Unlike chi-square values, which only indicate the difference between observed outcomes and random expectations, Ro/e values reveal whether specific cell clusters are enriched or depleted in particular tissues. A Ro/e value greater than 1 suggests that a cell cluster is more prevalent in a specific tissue than expected by chance, indicating enrichment. Conversely, a Ro/e value less than 1 indicates that a cell cluster is less prevalent than expected, suggesting depletion. By employing Ro/e calculations, researchers can effectively evaluate the tissue preferences of various cell clusters.

### Cell communication

The “CellChat” R package (version 1.5.0) is a tool designed to elucidate potential mechanisms of intercellular communication at the single-cell level. The function createCellChat generates a cellchat object, while the aggregateNet feature outlines the signaling outputs for each cell type. The netAnalysis_computeCentrality function calculates the input and output weights of specific signaling pathways. Data visualization is supported by the “CCPlotR” package, where the cc_heatmap function shows the frequency of intercellular communications, and the cc_arrow function illustrates receptor-ligand interactions relevant to target cells.

### Lactylation geneset score

Gene sets related to lactate were obtained from the MSigDB database (https://www.gsea-msigdb.org/). We calculated the lactylation gene set score using the AUCell package, specifically the AUCell_calcAUC function. This approach enabled us to compare lactate metabolism gene set scores across various cell types, including macrophage subclusters identified through secondary clustering, to identify cell subpopulations associated with lactate. We visualized the lactate metabolism gene set scores using ggplot2, presenting them as violin plots.

### Machine learning and prognostic model construction

Marker genes linked to the primary lactate-producing cell subtypes are identified using criteria of a log fold change (logFC) greater than 0.5 and a p-value less than 0.001. Core gene identification is performed using three machine learning methods: 1) LASSO regression with the “glmnet” package, 2) the CoxBoost algorithm via the “CoxBoost” package with an initial penalty of 1000, and 3) a Random Forest survival model through the “RandomForestRSF” package, using 1000 trees and evaluating gene significance with importance scores. All methods incorporate cross-validation. A Venn diagram analyzes the intersection of core genes identified by these algorithms to determine core risk prognostic genes. A multivariate regression analysis then develops the prognostic model. Risk scores are calculated by summing gene expression values weighted by their risk coefficients. GBM patients are classified into high-risk and low-risk groups based on the median risk score. Survival analyses and survival curves are generated using the “Survival” and “survminer” packages, while Receiver Operating Characteristic (ROC) curves are created with the “timeROC” package. The “ggplot2” package is used to produce Principal Component Analysis (PCA) plots, aiding in identifying key characteristics of the risk groups and enabling dimensionality reduction clustering.

### Enrichment analysis

Enrichment analysis includes Gene Ontology (GO), Kyoto Encyclopedia of Genes and Genomes (KEGG), and HALLMARK enrichment analyses. The clusterProfiler package is used for Gene Set Enrichment Analysis (GSEA) across these analyses, applying a threshold of an adjusted p-value below 0.05. For visualizing GO enrichment, the aPEAR package is used, while ggplot2 is applied for KEGG enrichment visualization. Additionally, the GseaVis package is utilized to visualize HALLMARK enrichment analysis.

### Immunology-related analysis

ssGSEA is a widely used method for evaluating immune cell types, focusing on 29 distinct categories and related immune processes. The “GSVA” and “GSEABase” packages calculate relative scores for these immune cells and processes. For visualization, the “ggpubr” and “introdataviz” packages create split violin plots. The “Estimate” package analyzes the tumor microenvironment, and correlation analyses explore relationships between risk scores and immune, stromal, and microenvironment scores. The “TCGAbiolinks” package retrieves single nucleotide polymorphism (SNP) data for GBM patients, while “maftools” calculates tumor mutational burden (TMB) scores for each patient. Additionally, the TIDE website (http://tide.dfci.harvard.edu/) assesses immune evasion and dysfunction status. Finally, “ggpubr” generates boxplots to depict the expression levels of immune checkpoint molecules.

### Drug sensitivity analysis

The pRRophetic algorithm was used to calculate drug sensitivity IC50 values, aiding in predicting patient responses to chemotherapeutic and targeted agents across different cohorts. By analyzing the correlation between risk scores and drug IC50 values, drugs with positive correlation coefficients emerged as potential therapeutic options for low-risk groups. In contrast, drugs showing a significant negative correlation with IC50 values were identified as potentially suitable for high-risk patients.

### Cell culture

Human brain astrocytes SVG p12 (FH0563) and glioblastoma cell lines U251 (FH0159) and U87 (FH0162) were obtained from Fuheng Biological Technology Co., Ltd. (Shanghai, China). These cells were cultured in DMEM medium (Gibco, Grand Island, NY, USA) supplemented with 10% fetal bovine serum (FBS; Gibco), 100 U/mL penicillin, and 100 μg/mL streptomycin (Invitrogen, Waltham, MA, USA). Cultivation occurred in a humidified incubator set at 37°C with 5% CO2.

### Cell transfection

The siRNA targeting CD93 was designed and synthesized by Tsingke Biotechnology Co., Ltd. (Beijing, China). Transfection was performed using Lipofectamine 3000 reagent (Invitrogen, Waltham, MA, USA) according to the manufacturer’s instructions. Post-transfection, cells were cultured at 37°C with 5% CO2 for 48 hours. The study comprised three experimental groups: cells transfected with si-CD93#1, si-CD93#2, and a negative control group (si-NC). The specific siRNA sequences used in the study were as follows:

si-CD93#1:GGCUACUGGUCUAUCGCAA;
si-CD93#2: GUGCAAGUUCAGCUUCAAA;

### Cell viability assay

Cell viability was assessed using the CCK-8 assay kit (Invigentech, IV08, USA). After 48 hours of siRNA transfection, U251 and U87 cell lines were seeded into 96-well plates at a density of 3,000 cells per well. At 24, 48, and 72 hours post-transfection, 10 μL of CCK-8 reagent was added to each well, followed by a 2-hour incubation at 37°C. The optical density (OD) at 450 nm was then measured with a microplate reader to determine the relative cell survival rate.

### Transwell invasion assay

The invasion assay employed Transwell chambers with 8μm pores (Corning, USA, Cat. No. 3422) placed in a 24-well plate. The upper chamber was coated with an 8-fold dilution of BD Matrigel. U251 and U87 cell lines, transfected with siRNA for 48 hours, were digested with trypsin and resuspended at 3 × 104^5 cells per chamber in 200 μL of serum-free medium. These cells were added to the upper chamber, while 600 μL of medium containing 20% fetal bovine serum (FBS) was placed in the lower chamber. The setup was incubated at 37°C with 5% CO2 for 48 hours. After incubation, non-migratory cells were removed from the upper chamber, and cells adhering to the membrane underside were fixed with 4% paraformaldehyde for 20 minutes. Following fixation, the cells were stained with crystal violet. Cell invasion was observed and documented using a microscope.

### Scratch assay

Forty-eight hours after siRNA transfection, U251 and U87 cell lines were re-plated into 6-well plates and cultured until they reached about 90% confluence within 24 hours. Using sterile 10 μl pipette tips, three linear scratches were made on the cell monolayer. The wells were then rinsed with phosphate-buffered saline (PBS) to remove any detached cells. A medium containing 4% fetal bovine serum (FBS) was added to each well, and the cells were further cultured. The wound healing process was observed, and images of the scratched regions were captured at 0 and 24 hours using a microscope.

### Quantitative real-time PCR (qRT-PCR)

U251 and U87 cells were collected 48 hours post-siRNA transfection. The procedure followed the kit’s instructions. Total RNA was extracted using the RNAiso Plus Reagent Kit (Takara, 9108/9109, Japan). mRNA was then reverse-transcribed into cDNA with the PrimeScript RT Reagent Kit (Takara, RR037A, Japan). Real-time quantitative PCR was conducted using the TB Green Premix Ex Taq II Reagent Kit (Takara, RR820A, Japan). GAPDH served as the normalization control, and CD93 mRNA expression levels were determined using the 2-ΔΔCt method. The primer sequences are as follows:

CD93-F: 5’-GGCAGACAGTTACTCCTGGGTT-3’,
CD93-R: 5’-GGAGTTCAAAGCTCTGAGGATGG-3’;
GAPDH-F：5’- ACAGCCTCAAGATCATCAGCA-3’;
GAPHD-R: 5’-ATGAGTCCTTCCACGATACCA-3’;

### Data analysis

Statistical analyses utilized R software (version 4.3.1). Continuous variables were analyzed with either Student’s t-test or the Mann-Whitney U test, while categorical variables were assessed using the chi-square test or Fisher’s exact test. Kaplan-Meier survival analysis estimated survival outcomes. For analyses with multiple comparisons, such as differences in clinical features across risk groups, P-values were adjusted for the false discovery rate using the Benjamini-Hochberg method. Statistical significance was denoted as follows: *P < 0.05, **P < 0.01, ***P < 0.001, and NS for no significant difference.

## Result

### The scRNA profiling of GBM

**Fig 1:**
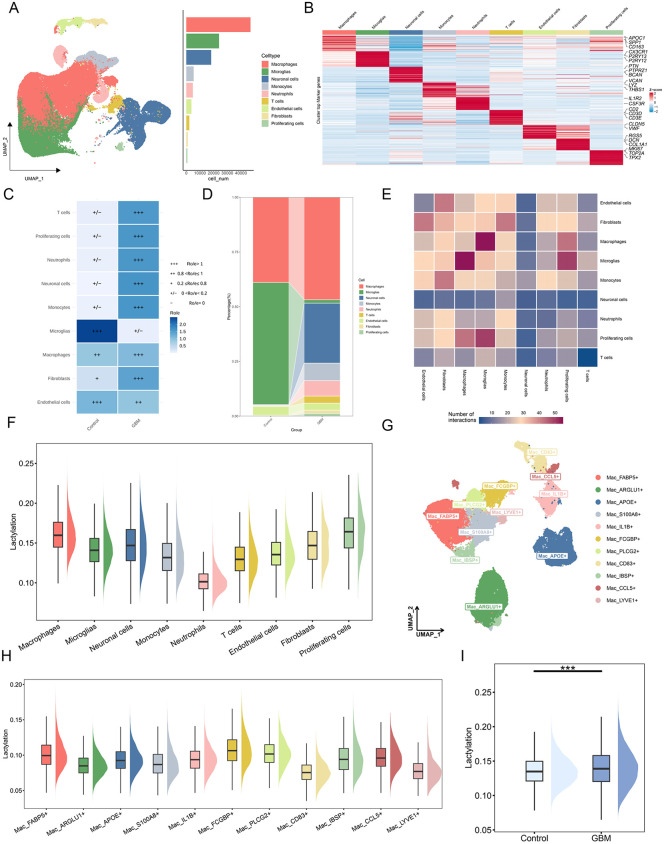
Immune landscape of single-cell data in GBM. **(A)** Identification of nine cell types in GBM. **(B)** Gene annotation for these nine cell types. **(C)** Distribution comparison of the nine cell types between control and GBM tissues. **(D)** R o/e scores for each of the nine cell types. **(E)** Heatmap illustrating communication among the nine cell types. **(F)** Lactylation gene set scores for the nine cell types. **(G)** Comparison of lactylation gene set scores between normal and GBM tissues. **(C)** UMAP plot depicting 11 cell subtypes following secondary clustering of macrophages. **(E)** Presentation of lactylation gene set scores between control and GBM tissues. **(F)** Lactylation gene set scores across 11 macrophage subtypes. **(G)** Identification of 11 macrophage subtypes in GBM. **(H)** Comparison of lactylation gene set scores between normal and GBM tissues. **(I)** Lactylation gene set scores among the 11 macrophage subtypes.

To thoroughly understand the cell types and molecular changes linked to GBM, we integrated two single-cell datasets (GSE141383 and GSE162631) and used the Harmony algorithm to correct for batch effects. After implementing stringent control measures, we analyzed 114,630 cells, including 45,282 control cells and 69,348 primary GBM cells. Unsupervised cell clustering was performed with a resolution parameter of 0.5, and we categorized these cells into nine distinct populations (Fig 1A). Specific annotations were based on markers for macrophages (OC1, SPP1, CD13), microglia (CX3CR1, P2RY12, P2RY13), neuron-like cells (PTN, PTPRZ, BCAN), monocytes (VCAN, LYZ, THBS1), neutrophils (IL12, CSF3R), T cells (CD2, CD3D, CD3E), endothelial cells (CLDN5, VWF), fibroblasts (RGS5, DCN, COLA1), and proliferating cells (MKI67, TOP2A, TPX2) (Fig 1B).To identify cell types closely associated with GBM progression, we conducted an observed-to-expected ratio (Ro/e) analysis, a method commonly used to assess tissue preference. Results indicated that microglia and endothelial cells were predominantly enriched in control tissues, while other cell types were concentrated in GBM tissues (Fig 1C). This finding suggests that tumorigenesis mobilizes various cells to collaboratively reshape the tumor microenvironment. We further evaluated the relative differences in cell types between GBM and control tissues. Notably, macrophages, neuron-like cells, monocytes, and neutrophils significantly increased in GBM, highlighting their critical roles in GBM development and progression (Fig 1D).Cell communication analysis revealed that macrophages and microglia exhibited the highest incoming and outgoing signal intensities (Fig 1E). Although increasing evidence suggests that excessive lactylation activation is closely associated with tumor progression, its impact on GBM progression remains unclear. We obtained a lactylation-related gene set from the MSigDB database and applied the AUCell score to evaluate the nine cell types. Macrophages had the highest lactylation gene set score (Fig 1F), leading us to speculate that macrophages may promote GBM progression through the lactylation pathway.Subsequently, we performed a secondary clustering analysis of macrophages and identified 11 different macrophage subtypes (Fig 1G). Interestingly, the FCGBP+ macrophage subtype showed the highest lactylation gene set score (Fig 1H). Additionally, we compared lactylation gene set scores between the control and GBM groups, finding that the GBM group had a significantly higher score (P < 0.01), further indicating lactylation as a important factor in GBM occurrence and development (Fig 1I).

### Machine learning construct FCGBP+ macrophages signatures

**Fig 2:**
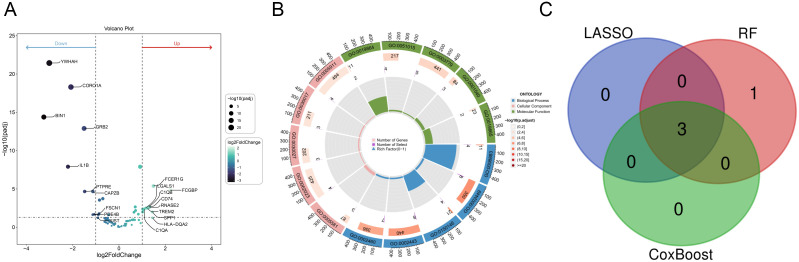
Construction of FCGBP+ macrophages prognostic genes. **(A)** The volcano plot illustrates the differentially expressed genes in FCGBP+ macrophages. **(B)** The GO enrichment analysis highlights the differentially expressed genes in FCGBP+ macrophages. **(C)** The Venn diagram shows the gene intersections identified by three distinct machine learning algorithms.

We identified marker genes for FCGBP+ macrophages using screening criteria of logFC > 0.5 and P < 0.001. We validated their expression in both control and GBM tissues from the TCGA-GBM cohort. Our analysis revealed 12 genes were upregulated and 9 were downregulated in GBM (Fig 2A). GO enrichment analysis of these differentially expressed genes indicated their involvement in immune cell regulation, cell differentiation, and other biological processes (Fig 2B). To pinpoint core genes of FCGBP+ macrophages, we employed three machine learning algorithms. The CoxBoost algorithm achieved the most stable model at the 117th boost, identifying three core genes with a partial log likelihood of 210.4623 (S1A Fig). LASSO regression, which allows for shrinkage and cross-validation, retained three highly reliable core genes with lambda.min = 0.1060313 (S1B and S1C Fig). The random forest survival model achieved the lowest error rate of 0.4783 at 143 trees, ranking differential genes by importance and identifying four genes with an importance score above zero (S1D and S1E Fig). We constructed a Venn diagram from the outputs of these algorithms and identified intersecting genes CD93, FCER1G, and GRB2 as core genes (Fig 2C).

### Validation of prognostic features and clinical value

**Fig 3:**
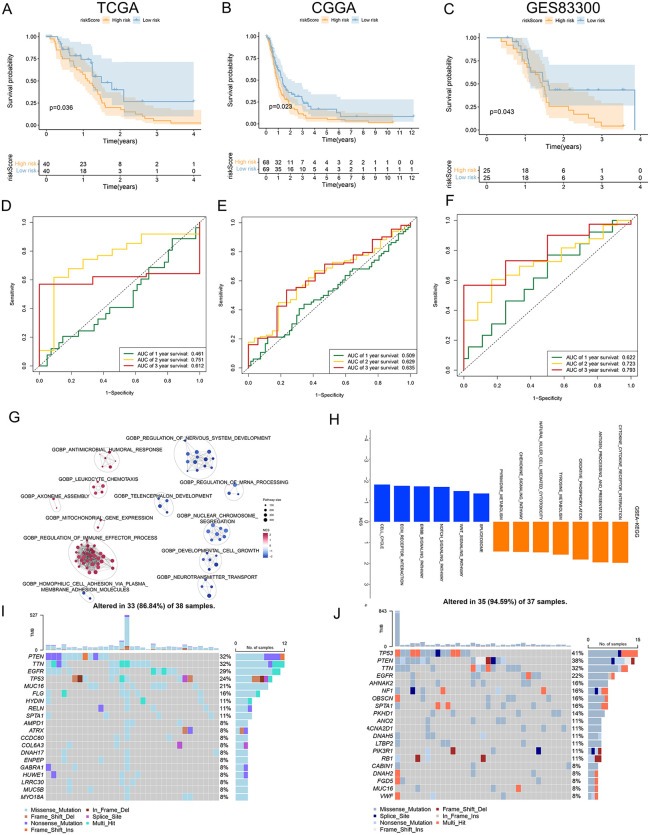
Validation and enrichment analysis of the risk prognostic model. **(A-C)** Kaplan-Meier survival curves illustrate overall survival (OS) for patients across various risk models within the TCGA, CGGA, and GSE83300 cohorts. **(D-F)** ROC curves depict 1-year, 2-year, and 3-year survival rates for different risk models in the TCGA, CGGA, and GEO GSE83300 datasets. **(G)** GSEA pathway analysis compares the high-risk and low-risk groups. **(H)** KEGG pathway analysis contrasts the high-risk and low-risk groups. (I-J) mutational landscape plot provide a summary of mutation rates in high-risk versus low-risk populations. (*p < 0.05; **p < 0.01; ***p < 0.001; NS: not statistically significant).

We developed a risk model based on three lactylation genes: CD93, FCER1G, and GRB2. Using Lasso analysis and multivariate Cox regression, we calculated a risk score for each GBM patient. Patients were then categorized into high-risk and low-risk groups according to the median risk score. In the training cohort (TCGA-GBM dataset) and two validation sets (CGGA dataset, GSE83300), we found a significant difference in overall survival between these groups. High-risk patients had lower survival rates compared to low-risk patients (P < 0.05) (Fig 3A–3C). To assess the prognostic power of these three genes for GBM, we performed time-dependent receiver operating characteristic (ROC) curve analyses in both the training and validation sets. The average area under the curve (AUC) of the risk model at 1, 2, and 3 years was 0.55, underscoring its biological significance (Fig 3D–3F). We also identified key biological processes and pathways that distinguished low-risk from high-risk patients. Gene Ontology (GO) enrichment analysis indicated that low-risk patients exhibited higher immune effector processes and increased activation and expression of mitochondrial genes (Fig 3G). Additionally, KEGG enrichment analysis revealed that pathways related to cytokine receptor interaction, antigen presentation, and oxidative phosphorylation were upregulated in the low-risk group, whereas cancer pathways such as WNT and NOTCH were downregulated (Fig 3H). These findings help explain the differences observed between the risk groups. The low-risk group showed a higher mutation frequency (94.59%), with TP53 being the most frequently mutated gene (Fig 3I and 3J). Conversely, the high-risk group had a mutation frequency of 86.84%, with PTEN mutations being predominant (Fig 3I and 3J).

### Immune-related features of the risk model

We evaluated immune infiltration across various risk categories and core genes using the ssGSEA algorithm to calculate scores for 29 distinct immune cell types and processes. Our results showed that the low-risk group had higher infiltration levels of dendritic cells (DCs), neutrophils, T helper cells, and tumor-infiltrating lymphocytes (TILs). This group also demonstrated enhanced T cell costimulation and type I interferon responses (S2A Fig). Furthermore, the core genes associated with the three models exhibited unique relationships with TNF family genes and chemokines. Notably, FCER1G had significant negative correlations with most TNF family genes and chemokines, whereas CD93 and GRB2 displayed partial positive correlations with these genes and chemokines (S2B and S2C Fig). To assess TME activity, we used TME scores and found that high-risk scores were significantly negatively correlated with both immune scores and TME scores. This finding suggests that lower risk scores may be linked to more immunologically active microenvironments and relatively lower tumor purity (S2D–S2F Fig).

### Immunotherapy efficacy stratification by immune microenvironment risk model

**Fig 4:**
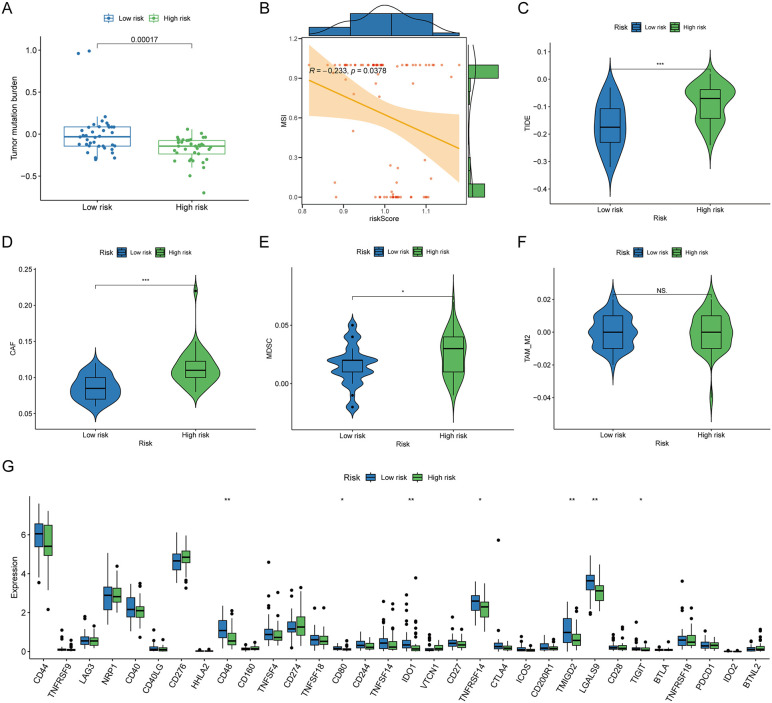
Characteristics of multiple immune microenvironment scoring systems in risk models. **(A)** Analysis of Immune Mutation Burden in High-Risk and Low-Risk Groups **(B)** Correlation Analysis of MSI Scores Between High-Risk and Low-Risk Groups.**(C)** TIDE Score Analysis in High-Risk and Low-Risk Groups **(D)** CAF Score Analysis in High-Risk and Low-Risk Groups **(E)** MDSC Score Analysis in High-Risk and Low-Risk Groups.**(F)** TAM_M2 Score Analysis in High-Risk and Low-Risk Groups.(G)Correlation Analysis of Immune Checkpoint-Related Molecules Between High-Risk and Low-Risk Groups.*p < 0.05; ** p < 0.01; *** p < 0.001;NS: not statistically significant.

We assessed the effectiveness of immunotherapy across various risk categories and found that the tumor mutation burden (TMB) was higher in the low-risk cohort. Additionally, a significant negative correlation emerged between microsatellite instability (MSI) and risk scores, indicating that low-risk patients exhibit elevated levels of both TMB and MSI, making them more suitable candidates for immunotherapy (Fig 4A and 4B). In contrast, the high-risk group demonstrated significantly increased TIDE scores and a higher prevalence of tumor-associated fibroblasts and myeloid-derived suppressor cells (MDSCs), suggesting a link between high-risk status and less favorable outcomes from immunotherapy (Fig 4C–4F). Our analysis further revealed that immune checkpoint molecules were predominantly expressed at higher levels in the low-risk group, reinforcing the idea that these patients are more likely to benefit from immunotherapy (Fig 4G). Consequently, individuals in the low-risk group may represent a promising demographic for immunotherapeutic strategies.

### Analysis of antitumor drugs in high-risk and low-risk groups

**Fig 5:**
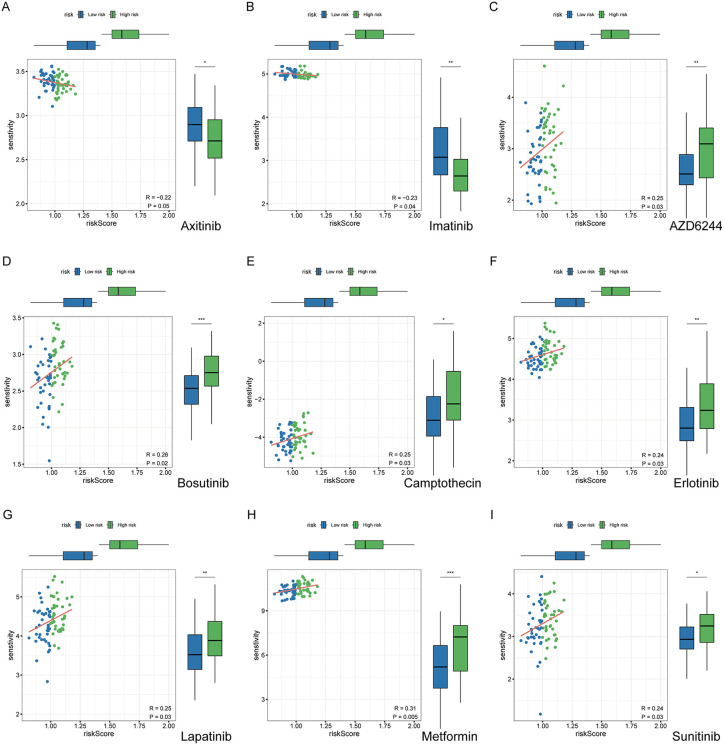
Predicted drug sensitivity stratified by immune microenvironment risk score. **(A–I)** The pRRophetic algorithm predicts correlations between nine clinically relevant antitumor drugs and drug sensitivity in patients with varying risk scores. **(A)** Axitinib, **(B)** Imatinib, **(C)** AZD6244, **(D)** Bosutinib, **(E)** Camptothecin, **(F)** Erlotinib, **(G)** Lapatinib, **(H)** Metformin, **(I)** Sunitinib. Box plots compare the distribution of predicted sensitivity scores between low-risk and high-risk groups, where *p < 0.05; ** p < 0.01; *** p < 0.001;NS: not statistically significant.

We performed a drug sensitivity prediction analysis using the pRRophetic algorithm to calculate IC50 values for patients in different risk groups. Axitinib and Imatinib demonstrated a significant negative correlation with the risk score, suggesting their potential effectiveness for high-risk patients (Fig 5A and 5B). In contrast, seven drugs, including AZD6244, showed a positive correlation with the risk score, indicating their suitability for low-risk patients. Among these, metformin exhibited the strongest and statistically significant correlation (Fig 5C–5I).

### CD93 is potentially a important gene in models linked to poor prognosis in GBM

**Fig 6:**
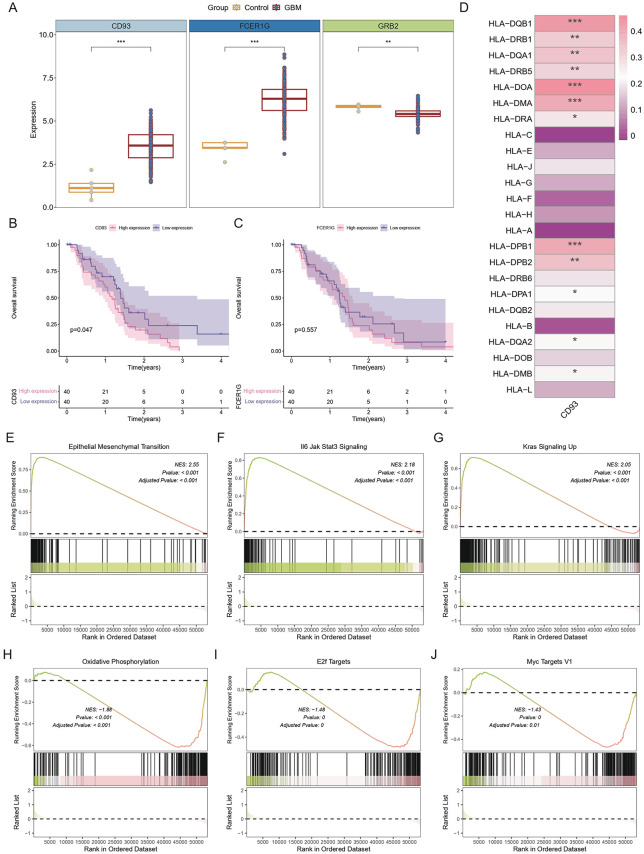
Prognostic model core gene screening. **(A)** Expression levels of CD93, FCER1G, and GRB2 in the TCGA-GBM cohort. **(B-C)** Survival curve analysis of CD93 and CER1G. **(D)** Correlation analysis of CD93 with human leukocyte antigen (HLA) family genes.(E-J)KEGG pathway enrichment analysis of CD93-related genes. (*p < 0.05; ** p < 0.01; *** p < 0.001;NS: not statistically significant).

To identify key molecular biomarkers for our prognostic model, we evaluated the expression levels of three critical genes in GBM. Our analysis showed that only CD93 and FCER1G were significantly upregulated in GBM tissues, indicating their potential roles in tumor progression (Fig 6A). Kaplan-Meier survival analysis further demonstrated that CD93 was the sole gene with a statistically significant impact (P < 0.05) on GBM patient survival. Higher CD93 expression correlated with poorer overall survival outcomes (Fig 6B and 6C). Thus, CD93 emerged as a promising molecular biomarker for GBM. Interestingly, CD93 did not significantly affect the tumor antigen presentation process but showed a positive correlation with HLA family molecules (Fig 6D), suggesting involvement in alternative tumor-promoting mechanisms. Additionally, hallmark enrichment analysis compared pathway variations between groups with high and low CD93 expression. Patients with elevated CD93 expression showed increased activity in the epithelial-mesenchymal transition (EMT), interleukin-6 (IL6), and KRAS pathways. Conversely, those with lower CD93 expression exhibited upregulation of processes such as oxidative phosphorylation (Fig 6E–6J). These pathways may elucidate how CD93 contributes to GBM progression.

### CD93 is upregulated in GBM cell lines and enhances GBM cell proliferation, invasion, and migration

**Fig 7:**
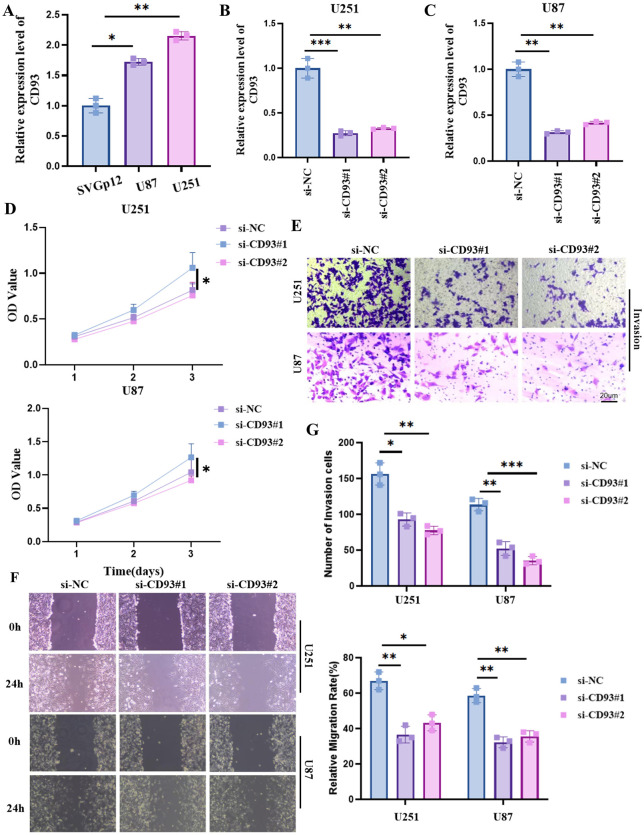
Validation of the role of core gene CD93 in cytology experiments. **(A)** Expression levels of CD93 mRNA in GBM cell lines. **(B-C)** Knockdown efficiency of CD93 mRNA in U251 and U87 cells. **(D)** CCK8 analysis showed that interfering with CD93 expression significantly inhibited the proliferation ability of U251 and U87 cells. **(E-F)** Interfering with CD93 expression significantly inhibited the migration and invasion abilities of U251 and U87 cells. **(G)** Statistical graphs of invasion and migration experiments (*p < 0.05, **p < 0.01, ***p < 0.001, ns: not significant).

In summary, the findings suggest that CD93 is a key gene linked to poor prognosis in GBM. Quantitative reverse transcription polymerase chain reaction (qRT-PCR) analyses revealed that human astrocytoma cells exhibited higher CD93 expression levels compared to human brain astrocytes (Fig 7A). We transfected siRNA#1 and #2 into U251 and U87 cell lines and confirmed the transfection efficiency of CD93 (Fig 7B and 7C). Cell Counting Kit-8 (CCK8) assays showed that CD93 knockdown significantly inhibited the proliferation of both U251 and U87 cells (Fig 7D). Furthermore, scratch and Transwell assays indicated that CD93 knockdown reduced the migratory and invasive capabilities of U251 and U87 cells. Overall, these results imply that CD93 functions as a positive regulator in the context of GBM.

## Discussion

This study innovatively integrates single-cell sequencing data with extensive transcriptome datasets from public databases to explore lactic acid metabolism in macrophages within the GBM tumor microenvironment. We conducted a comprehensive analysis of GBM by incorporating large-scale transcriptome data. Using single-cell sequencing, machine learning, and immune infiltration analysis, we examined the effects of lactation modification on GBM. Our findings indicate that genes associated with lactation modification could serve as novel therapeutic targets. Additionally, we developed a patient survival prognosis model with moderate predictive ability.The significance of lactation modification in the glioblastoma tumor microenvironment is increasingly recognized, with lactate dehydrogenase (LDH) identified as a major prognostic marker for this malignant tumor [16]. Tumor cells often undergo metabolic reprogramming, leading to excessive lactic acid production, which promotes angiogenesis, immune evasion, and drug resistance [17–18]. Research by Yan et al. [19] confirmed that lactic acid enhances GPR65 expression on tumor-associated macrophages (TAMs), activating the cAMP/PKA/CREB signaling pathway, thereby facilitating glioblastoma invasion and metastasis. Consequently, lactic acid modification presents a promising target for GBM treatment.Further research is essential to identify specific targets related to lacylation, which could become new therapeutic targets for GBM. Developing innovative therapeutic strategies based on this metabolic pathway is crucial.

We obtained RNA expression profile data for GBM from the TCGA database, while gene mutation data and clinical information were sourced from the GEO and CGGA databases. This comprehensive dataset facilitated an in-depth analysis of GBM’s molecular characteristics. Additionally, we utilized single-cell RNA sequencing data to examine GBM heterogeneity within the tumor microenvironment, identifying nine distinct cell types: macrophages, microglia, neuron-like cells, monocytes, neutrophils, T cells, endothelial cells, fibroblasts, and proliferation-like cells. These findings offer valuable insights into the mechanisms underlying GBM occurrence.We applied the lactic acid metabolism gene set score to identify individual cells involved in lactic acid metabolism. Macrophages exhibited the highest gene set score, consistent with Yang et al.’s findings [20], which reported a significant link between lactic acid metabolism and gastric cancer progression, with macrophages showing the highest infiltration levels. This supports our results. To identify the primary cellular subtypes influencing lactic acid metabolism, we conducted secondary dimensionality reduction clustering, classifying macrophages into 11 subtypes. The FCGBP+ macrophage subtype displayed the highest lactate metabolism gene set score.Subsequently, we analyzed core genes related to the FCGBP+ macrophage subtype in the TCGA-GBM cohort, identifying 12 up-regulated and 9 down-regulated genes in GBM. These differentially expressed genes are involved in immune cell regulation, cell differentiation, and various biological pathways. Our research enhances the understanding of lactic acid metabolic activity variations in GBM cells.The primary objective of this study was to establish a robust prognostic model for GBM. Using machine learning algorithms, we constructed a predictive model based on key lactic acid metabolism-related genes. This model effectively stratifies patients into high-risk and low-risk groups. Survival analysis revealed that high-risk patients have shorter prognoses and survival times. Notably, the risk model, validated across the TCGA training set, CGGA validation set, and GEO validation set, achieved an average area under the curve (AUC) of over 0.6 at 1, 2, and 3 years. This demonstrates the model’s stability and accuracy in multi-cohort studies and its potential clinical application.Furthermore, our study conducted an in-depth analysis of the immune characteristics of GBM, revealing significant differences between high-risk and low-risk groups in immune cell infiltration, immune checkpoint gene expression, and tumor mutation burden. These findings suggest that the prognostic model’s risk stratification mechanism may reflect immunological differences among GBM tumors in various populations, highlighting the need to explore differential immunotherapy responses among patient groups.

Through the analysis of core lactate-promoting genes, we identified CD93 as a key gene associated with poor prognosis in GBM. Polymeric protein-2 (MMRN2), a widely expressed extracellular matrix protein, interacts specifically with CD93, enhancing endothelial cell adhesion and migration, thereby facilitating angiogenesis [21]. Moreover, CD93 is significantly upregulated in breast cancer, where its interaction with MMRN2 activates integrin β1, promoting tumor growth and angiogenesis through the PI3K/AKT/SP2 signaling pathway [22]. Consequently, we propose that CD93 could serve as a potential biomarker for GBM. Cell experiments demonstrated high CD93 expression in GBM cells, and inhibiting CD93 expression significantly reduced the proliferation, invasion, and migration abilities of U87 and U251 cells. In this study, we identified key lactylation-related genes using single-cell RNA sequencing technology and developed a prognostic model. We revealed the mechanisms of lactate-mediated metabolic and immune escape in GBM and identified potential biomarkers and therapeutic targets. However, further validation and efficacy evaluation are necessary.

This study has several limitations that require objective explanation. First, the model is at risk of overfitting. The sample size and inclusion of multi-dimensional features make it prone to capturing noise and individual differences within the training cohort, which may reduce its ability to generalize to external populations. To address overfitting, we employed hierarchical cross-validation, retained clinically meaningful core features to minimize redundancy, and constrained model complexity and hyperparameter settings. Furthermore, the model’s AUC is only at a medium level, indicating limited discriminative efficacy and lacking fully reliable clinical application value. Future enhancements in predictive performance could be achieved by optimizing feature combinations and increasing the sample size.

## Supporting information

S1 Fig Screening and identification of hub prognostic genes using multiple machine learning algorithms. (A – E) Flowcharts of three machine learning screening and modeling processes.
(PNG)

S2 Fig Immunological landscape characterization and correlation analysis of the risk signature. (A) Differences in immune molecule infiltration between high-risk and low-risk groups. (B) Correlation analysis between three core genes and TNF family genes. (C) Correlation analysis between three core genes and chemokine family genes. (D-F) Correlation analysis between risk score and ImmuneScore,StromalScore,ESTIMATEScore.(*p < 0.05;**p < 0.01;***p < 0.001;NS: not statistically significant).
(PNG)

S3 Fig Cellular heterogeneity, trajectory, expression signature, pathway enrichment and cell composition analysis of the risk subgroups. (A) Single-cell sequencing classified glioblastoma multiforme (GBM) into 21 clusters. (B) Key ligand-receptor pairs for input and output signals of macrophages and microglia. (C) Scatter plot of key genes for 11 macrophage subtypes. (D) tSNE2 patterns of different risk models in the TCGA, CGGA, and GSE83300 cohorts. (E) KEGG pathway analysis between the high-risk and low-risk groups. (F) Line graph showing the proportion of total macrophages in GBM. (G) Proportion of FCGBP+ macrophages in GBM. (H) Proportion of CD93 + macrophages in GBM.
(PNG)

## Abbreviation:

GBM: Glioblastoma
TNF: Tumor Necrosis Factor
TME: Tumor Microenvironment
TAM: Tumor-Associated Macrophage
CAF: Cancer-Associated Fibroblast
EC: Endothelial Cell
scRNA-seq: Single-cell RNA Sequencing
GEO: Gene Expression Omnibus database
TCGA: The Cancer Genome Atlas database
AUC: Area Under the Curve
ROC: Receiver Operating Characteristic
