# Supplementary figures and images for: Integrated single-cell RNA sequencing and Bulk-RNA technologies reveal the immunological characteristics of lactylation related-genes in glioblastoma
Source: PLoS One. 2026 Jun 26;21(6):e0351849. doi: 10.1371/journal.pone.0351849 (PMC13308864; doi:10.1371/journal.pone.0351849)

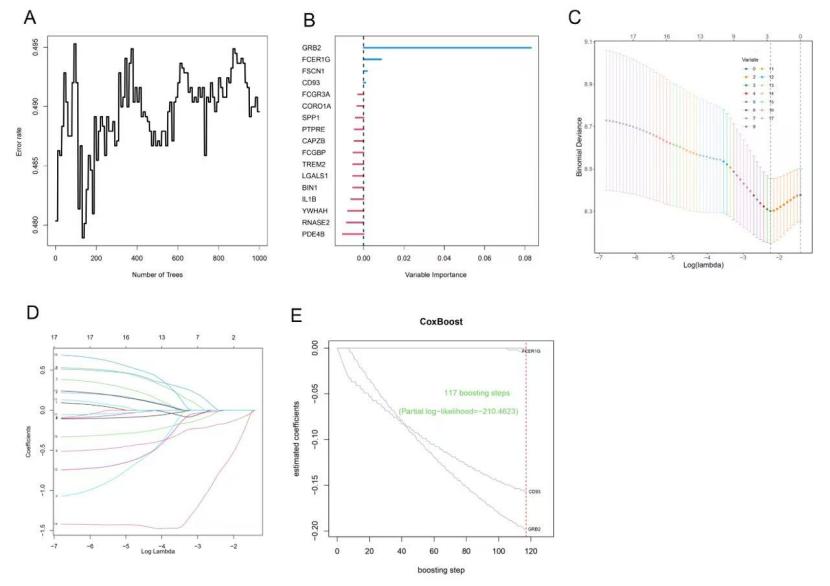

Supplement: S1 Fig — (A – E) Flowcharts of three machine learning screening and modeling processes. (PNG) [file pone.0351849.s001.png]

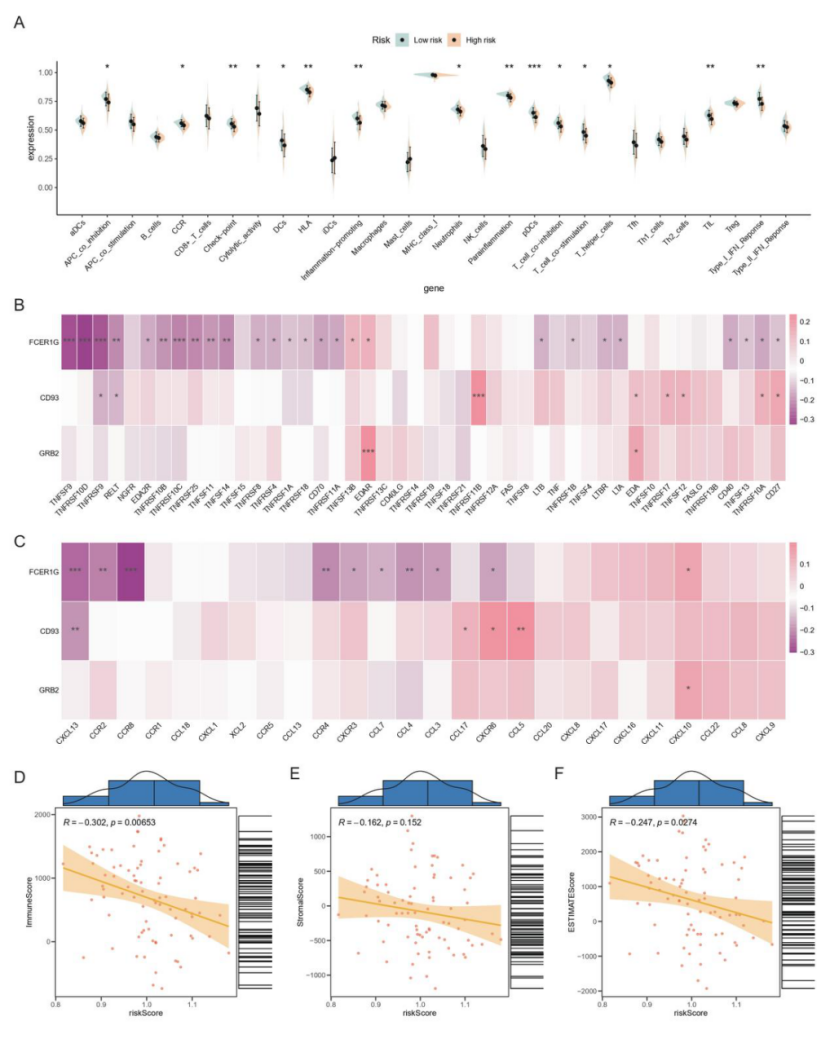

Supplement: S2 Fig — (A) Differences in immune molecule infiltration between high-risk and low-risk groups. (B) Correlation analysis between three core genes and TNF family genes. (C) Correlation analysis between three core genes and chemokine family genes. (D-F) Correlation analysis between risk score and ImmuneScore,StromalScore,ESTIMATEScore.(*p < 0.05;**p < 0.01;***p < 0.001;NS: not statistically significant). (PNG) [file pone.0351849.s002.png]

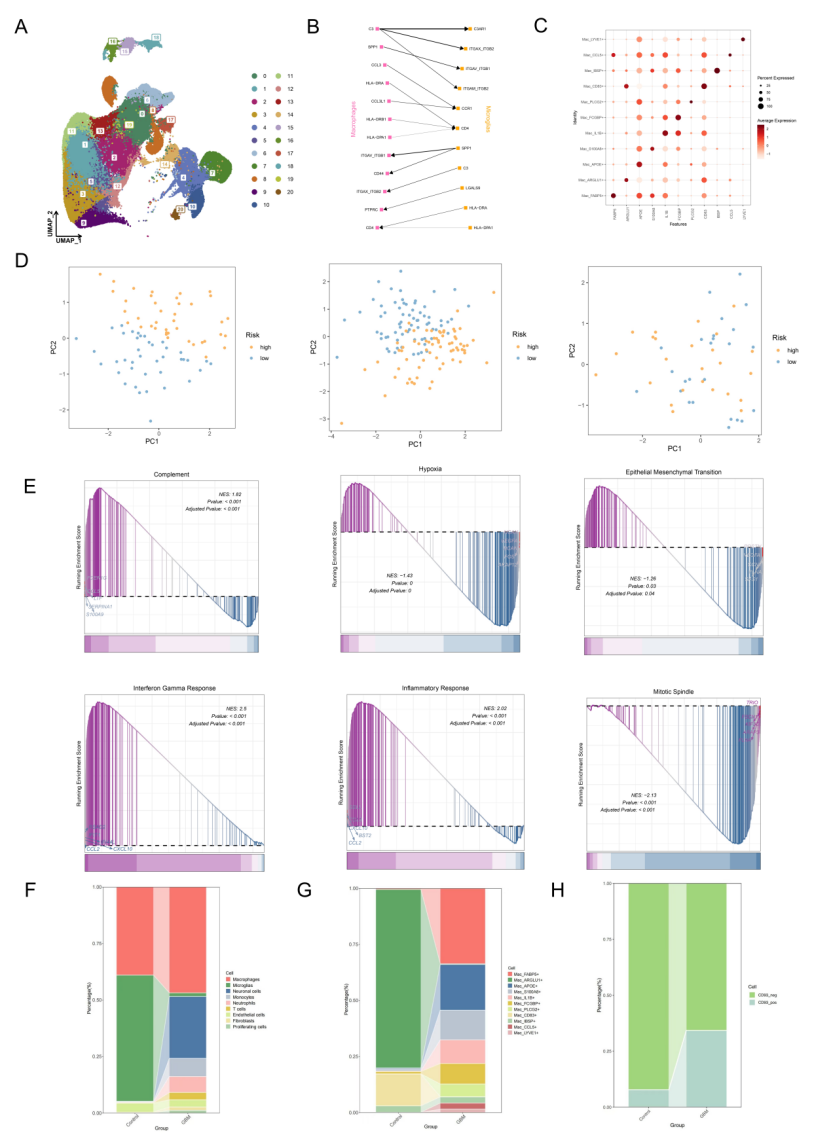

Supplement: S3 Fig — (A) Single-cell sequencing classified glioblastoma multiforme (GBM) into 21 clusters. (B) Key ligand-receptor pairs for input and output signals of macrophages and microglia. (C) Scatter plot of key genes for 11 macrophage subtypes. (D) tSNE2 patterns of different risk models in the TCGA, CGGA, and GSE83300 cohorts. (E) KEGG pathway analysis between the high-risk and low-risk groups. (F) Line graph showing the proportion of total macrophages in GBM. (G) Proportion of FCGBP+ macrophages in GBM. (H) Proportion of CD93 + macrophages in GBM. (PNG) [file pone.0351849.s003.png]
